# Application of Nanomedicine in Immunotherapy: Recent Advances and Prospects

**DOI:** 10.3390/pharmaceutics15071910

**Published:** 2023-07-09

**Authors:** João Paulo Figueiró Longo, Luis Alexandre Muehlmann

**Affiliations:** 1 Department of Genetics and Morphology, Institute of Biological Sciences, University of Brasília, Brasilia 70910-900, Brazil; 2 Faculty of Ceilândia, University of Brasilia, Brasilia 72220-275, Brazil; luismuehlmann88@gmail.com

Nanomedicine is a special medical field focused on the application of nanotechnology to provide innovations for healthcare in different areas, including the treatment of a wide variety of diseases, including cancer [1,2], infections [3,4], and auto-immune disorders. The field emerged during the 1980s, aligning with the approval of the first regulatory-agency-approved nanomedical oncological drugs [2,5]. Additionally, nanotechnology has played a pivotal role in the development of mRNA vaccines utilized during the COVID-19 pandemic [6], further establishing its enduring significance in the domains of science and biomedical innovation.

The reasons for the use of nanotechnology in biomedicine can vary but are mostly the protection and/or delivery of bioactive molecules to target tissues. The general idea is to create nanoscopic platforms that can interact differently with biological systems, either through pharmacokinetic modifications or through the preferential activation of some biological pathway [5,7]. 

The intricate biomolecular interactions that underlie the functioning of the biological system take place within the nanoscale. So, nanoparticles can be designed to be similar in size to key biological structures like large biomolecules or small organelles, enabling them to interact with biological systems in unique ways, which can improve the effects of drugs used in the treatment of certain diseases. In other words, highly distinct and improved biological effects can emerge from such interactions [8,9]. 

For this Special Issue, we sought submissions specifically focusing on the utilization of nanomedicine in various types of immunotherapies. In terms of quantity, a total of ten papers were published, six of them original articles and four review articles. The contributions were made by a diverse group of sixty-nine authors hailing from eight countries: Austria, Brazil, China, France, Italy, Japan, Korea, and the United States of America. Currently, the published articles have been accessed by nearly twenty thousand readers.

Regarding the topics, Chen et al. (2023) [10], Sasaki et al. (2022) [11], and Lodeserto et al. (2022) [12] applied nanotechnology to deliver different types of active molecules to target. Chen et al. (2023) [10] reported the development of an innovative polypeptide hydrogel to deliver immune checkpoint inhibitors and doxorubicin to improve tumor immunotherapy. As the main results, the authors observed a significant reduction in tumor recurrence by using this immunotherapy strategy. Following the delivery strategy, Sasaki et al. (2022) [11] described the use of lipid nanoparticles to deliver mRNA for dendritic cells. With this strategy, the authors showed a therapeutic antitumor effect against a pre-clinical tumor model, and, Lodeserto et al. (2022) [12] reported the preparation of a spermidine nanocarrier to target specific cells. The authors showed that spermidine can activate immune cells, and this feature is related to immunosuppression reversion in the tumor environment. 

Following an immune activation strategy, Punz et al. (2022) [13] proposed the utilization of silica nanoparticles as platforms for delivering immune activation molecules. The authors demonstrated the potential of these platforms in modulating antigen uptake and promoting the maturation of antigen-presenting cells. Following a similar immune activation direction, Rodrigues et al. (2022) [14] proposed the use of phthalocyanine nanoemulsions to induce immunogenic cell death and exploit these cells as a platform for immune activation. Through this approach, the authors demonstrated that PDT-treated cancer cells used as a vaccine hinder tumor growth in pre-clinical models. 

Following the opposite direction, Lasola et al. (2021) [15] published an intriguing paper assessing the impact of various nanoparticles’ surface chemistry on mitigating undesired immune system activation. They aimed to observe an anti-inflammatory immune response. This approach holds potential for employing nanomaterials in the management of severe inflammatory diseases, such as sepsis.

Regarding the review articles included in this Special Issue, there were a total of four publications. Dias et al. (2022) [16] reported an interesting review collating information related to the use of iron oxide for immunotherapy for oncology applications. Rodrigues et al. (2022) [17] and Seong and Kim (2022) [18] published two reviews reporting the use of cell lysates to trigger the immune system. Rodrigues focused on the aspects related to immunogenic cell death, while Seong included more general cell lysates, including necrotic-induced cell death protocols. Lastly, Nigam et al. (2022) [19] reported an important review presenting an update on the use of nanotechnology in the immunotherapy of type 1 diabetes. 

In summary, this Special Issue presents a compilation of groundbreaking research papers and comprehensive reviews that delve into various dimensions of immunotherapy and nanomedicine. The invaluable insights shared in this publication offer profound insights into the potential of nanotechnology to enhance the efficacy of diverse immunotherapeutic modalities. We are confident that the wealth of high-quality information disseminated in this Issue will captivate the attention of both researchers and individuals seeking alternative cancer treatments, particularly those intrigued by the prospects of nanotechnological approaches for immunotherapies.

## References

[1] Faria R.S., de Lima L.I., Bonadio R.S., Longo J.P.F., Roque M.C., de Matos Neto J.N., Moya S.E., de Oliveira M.C., Azevedo R.B. (2021). Liposomal paclitaxel induces apoptosis, cell death, inhibition of migration capacity and antitumoral activity in ovarian cancer. Biomed. Pharmacother..

[2] Longo J.P.F., Muehlmann L.A., Calderón M., Stockmann C., Azevedo R.B. (2021). Nanomedicine in Cancer Targeting and Therapy. Front. Oncol..

[3] Mengarda A.C., Iles B., Longo J.P.F., de Moraes J. (2022). Recent trends in praziquantel nanoformulations for helminthiasis treatment. Expert Opin. Drug Deliv..

[4] Mengarda A.C., Iles B., Longo J.P.F., de Moraes J. (2022). Recent approaches in nanocarrier-based therapies for neglected tropical diseases. Wiley Interdiscip. Rev. Nanomed. Nanobiotechnol..

[5] Figueiro Longo J.P., Muehlmann L.A. (2020). Nanomedicine beyond tumor passive targeting: What next?. Nanomedicine.

[6] Figueiró Longo J.P., Muehlmann L.A. (2021). How has nanomedical innovation contributed to the COVID-19 vaccine development?. Nanomedicine.

[7] Anselmo Joanitti G., Ganassin R., Correa Rodrigues M., Paulo Figueiro Longo J., Jiang C.-S., Gu J., Milena Leal Pinto S., de Fatima M.A.d.S., Bentes de Azevedo R., Alexandre Muehlmann L. (2017). Nanostructured Systems for the Organelle-specific Delivery of Anticancer Drugs. Mini Rev. Med. Chem..

[8] Coelho J.M., Camargo N.S., Ganassin R., Rocha M.C.O., Merker C., Böttner J., Estrela-Lopis I., Py-Daniel K.R., Jardim K.V., Sousa M.H. (2019). Oily core/amphiphilic polymer shell nanocapsules change the intracellular fate of doxorubicin in breast cancer cells. J. Mater. Chem. B.

[9] Ganassin R., Horst F.H., Camargo N.S., Chaves S.B., Morais P.C., Mosiniewicz-Szablewska E., Suchocki P., Figueiro Longo J.P., Azevedo R.B., Muehlmann L.A. (2018). Selol nanocapsules with a poly (methyl vinyl ether-co-maleic anhydride) shell conjugated to doxorubicin for combinatorial chemotherapy against murine breast adenocarcinoma in vivo. Artif. Cells Nanomed. Biotechnol..

[10] Chen Z., Rong Y., Ding J., Cheng X., Chen X., He C. (2023). Injectable Polypeptide Hydrogel Depots Containing Dual Immune Checkpoint Inhibitors and Doxorubicin for Improved Tumor Immunotherapy and Post-Surgical Tumor Treatment. Pharmaceutics.

[11] Sasaki K., Sato Y., Okuda K., Iwakawa K., Harashima H. (2022). mRNA-Loaded Lipid Nanoparticles Targeting Dendritic Cells for Cancer Immunotherapy. Pharmaceutics.

[12] Lodeserto P., Rossi M., Blasi P., Farruggia G., Orienti I. (2022). Nanospermidine in Combination with Nanofenretinide Induces Cell Death in Neuroblastoma Cell Lines. Pharmaceutics.

[13] Punz B., Johnson L., Geppert M., Dang H.-H., Horejs-Hoeck J., Duschl A., Himly M. (2022). Surface Functionalization of Silica Nanoparticles: Strategies to Optimize the Immune-Activating Profile of Carrier Platforms. Pharmaceutics.

[14] Rodrigues M.C., de Sousa Júnior W.T., Mundim T., Vale C.L.C., de Oliveira J.V., Ganassin R., Pacheco T.J.A., Vasconcelos Morais J.A., Longo J.P.F., Azevedo R.B. (2022). Induction of Immunogenic Cell Death by Photodynamic Therapy Mediated by Aluminum-Phthalocyanine in Nanoemulsion. Pharmaceutics.

[15] Lasola J.J.M., Cottingham A.L., Scotland B.L., Truong N., Hong C.C., Shapiro P., Pearson R.M. (2021). Immunomodulatory Nanoparticles Mitigate Macrophage Inflammation via Inhibition of PAMP Interactions and Lactate-Mediated Functional Reprogramming of NF-κB and p38 MAPK. Pharmaceutics.

[16] Dias A.M.M., Courteau A., Bellaye P.-S., Kohli E., Oudot A., Doulain P.-E., Petitot C., Walker P.-M., Decréau R., Collin B. (2022). Superparamagnetic Iron Oxide Nanoparticles for Immunotherapy of Cancers through Macrophages and Magnetic Hyperthermia. Pharmaceutics.

[17] Rodrigues M.C., Morais J.A.V., Ganassin R., Oliveira G.R.T., Costa F.C., Morais A.A.C., Silveira A.P., Silva V.C.M., Longo J.P.F., Muehlmann L.A. (2022). An Overview on Immunogenic Cell Death in Cancer Biology and Therapy. Pharmaceutics.

[18] Seong J., Kim K. (2022). Activation of Cellular Players in Adaptive Immunity via Exogenous Delivery of Tumor Cell Lysates. Pharmaceutics.

[19] Nigam S., Bishop J.O., Hayat H., Quadri T., Hayat H., Wang P. (2022). Nanotechnology in Immunotherapy for Type 1 Diabetes: Promising Innovations and Future Advances. Pharmaceutics.

